# Identification of RNA Oligonucleotides Binding to Several Proteins from Potential G-Quadruplex Forming Regions in Transcribed Pre-mRNA

**DOI:** 10.3390/molecules201119733

**Published:** 2015-11-23

**Authors:** Taiki Saito, Wataru Yoshida, Tomomi Yokoyama, Koichi Abe, Kazunori Ikebukuro

**Affiliations:** 1 Department of Biotechnology and Life Science, Graduate School of Engineering, Tokyo University of Agriculture & Technology, 2-24-16 Naka-cho, Koganei, Tokyo 184-8588, Japan; 50013831103@st.tuat.ac.jp (T.S.); tyokoyama472@gmail.com (T.Y.); abe79kou@gmail.com (K.A.); 2 School of Biotechnology and Bioscience Tokyo University of Technology, 1404-1 Katakura-cho, Hachioji, Tokyo 192-0982, Japan; yoshidawtr@stf.teu.ac.jp

**Keywords:** RNA aptamer, G-quadruplex, first intron, 5′ UTR, heparin-binding proteins

## Abstract

G-quadruplexes (G4s) are noncanonical DNA/RNA structures formed by guanine-rich sequences. Recently, G4s have been found not only in aptamers but also in the genomic DNA and transcribed RNA. In this study, we identified new RNA oligonucleotides working as aptamers by focusing on G4-forming RNAs located within the pre-mRNA. We showed that the G4 in the 5′ UTR and first intron of VEGFA bound to the protein encoded in VEGFA gene, VEGF165, with high affinity. Moreover, G4-forming RNAs located within the PDGFA and the PDGFB introns bound to PDGF-AA and PDGF-BB, respectively, indicating that G4 in the pre-mRNA could be an aptamer. It had been reported that the putative G4-forming RNA sequences are located in some parts of most genes, thus our strategy for aptamer identification could be applicable to other proteins. It has been reported that some G4-forming RNAs in 5′ UTRs are involved in translation control; however, G4-forming excised intronic RNA function has not been revealed previously. Therefore, these findings could not only contribute to the identification of RNA aptamers but also provide new insights into the biological functioning of G4-forming RNAs located within intronic RNA sequences.

## 1. Introduction

RNAs fold into specific complex three-dimensional structures to provide catalytic and molecular recognition abilities. These functional RNAs have been identified in both natural and artificial RNAs [1]. Artificial functional RNAs that possess molecular recognition ability are known as RNA aptamers, and such aptamers can be selected from random RNA libraries by systematic evolution of ligands by exponential enrichment (SELEX) [2,3]. The chemically modified RNA aptamers are valuable as drug and molecular recognition elements in biosensors [4]. Indeed, the chemically modified RNA aptamers against vascular endothelial growth factor 165 (VEGF165) have been approved for the treatment of neovascular age-related macular degeneration [5]. Moreover, RNA aptamers have been utilized to develop ligand-inducible gene expression systems in synthetic biology [6,7,8].

SELEX enables the identification of aptamers against small molecules, peptides, proteins, and whole cells, including non-immunogenic and toxic targets [9]. Modified SELEX methods have been developed to efficiently select aptamers [10]. However, SELEX is labor-intensive and generally requires several months to obtain aptamers. Non-SELEX aptamer identification methods such as the *in silico* selection of aptamers have been reported [11]. Although such virtual screening methods are potentially powerful, an improvement in the system is required to enable the selection of an appropriate aptamer without additional *in vitro* selection. Aptamers can also be identified from genomic information without *in vitro* selection. Riboswitches, which sense metabolites to control gene expression, translation, splicing, and RNA stability, are known to be natural aptamers [1,12]. Riboswitch candidates have been identified by bioinformatic searches; however, these are limited to aptamers against small molecules [13].

We have previously reported that G4-forming DNAs that are located within the gene promoter region of a target protein may act as DNA aptamers against the target proteins [14]. The DNA aptamer identification method was designated as G4 promoter-derived aptamer selection (G4PAS). Using G4PAS, DNA aptamers against VEGF165, platelet-derived growth factor-AA (PDGF-AA), and retinoblastoma 1 (RB1) were identified. These aptamers bound to their respective target proteins with high affinity. In addition, the PDGF-AA and RB1 aptamers bound to VEGF165. However, the PDGF-AA and RB1 aptamers did not bind to VEGF121, which does not possess a heparin-binding domain, indicating that they bind to VEGF165 probably due to recognition of the heparin-binding domain.

We considered the G4 structure as a good scaffold for target protein recognition because G4 has twice the negative charge density of double helices [15] to interact with the cationic domain of the protein. Moreover, we have suggested that G4 tends to preferentially bind to the β-structures of proteins [16]. Therefore, we investigated whether G4-forming RNA aptamers could be obtained from the transcribed RNA of target proteins. It has been reported that potential G4-forming sequences are enriched at the transcription start site, the 5′ UTR, and in the first intron, which is on the non-template strand in the human genome [17]. This suggests that G4-forming RNAs mainly exist within the 5′ UTR and the first intronic region of RNA. We chose to use VEGF165, PDGF-AA, and PDGF-BB as target proteins since each of them contains a heparin-binding domain, and G4-forming sequences are enriched in these genes. Analyses of the binding of target proteins to the synthetic G4-forming RNAs located within the 5′ UTR or the first intronic RNA region of these target genes were performed *in vitro*.

## 2. Results and Discussion

### 2.1. Identification of G4-Forming RNA Sequences from VEGFA, PDGFA, and PDGFB Pre-mRNA Sequence

First we obtained several G4-forming RNA sequences from pre-mRNA of *VEGFA* by QGRS Mapper [18]. The *VEGFA* first intron contains eight putative G4-forming RNA regions (G_≥3_N_≥1_G_≥3_N_≥1_G_≥3_N_≥1_G_≥3_; length ≤ 35-mer). Of these putative G4 RNAs, two G4 RNAs (VEGFA intronic G4_1 and G4_2) were randomly selected and chemically synthesized. Then we investigated the binding abilities of synthesized RNA that had putative G4 structures (Table 1). The genomic positions of the RNAs are illustrated in Supplemental Figure S1.

In addition, it has been reported that the 5′ UTR of VEGFA mRNA contains an 18-mer G4-forming RNA that is involved in internal ribosomal entry site (IRES)-mediated translation initiation [19]. Therefore, that 18-mer G4-forming RNA of VEGFA was chemically synthesized and binding analysis was performed (VEGFA 5′ UTR G4, Table 1).

### 2.2. Binding Assay of G4 RNAs Derived from VEGFA Transcript to VEGF165

**Figure 1:**
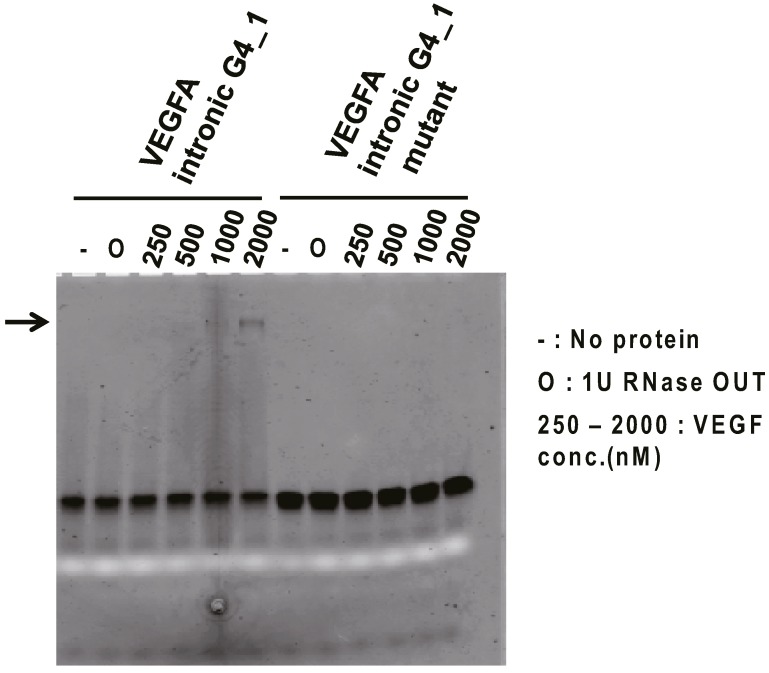
Electromobility shift binding assay (EMSA) of VEGFA intronic G4_1 to VEGF165. In the presence or absence of targets, fluorescein-labeled VEGFA intronic G4_1 (or its mutant) were electrophoresed on 15% polyacrylamide gel in TBE buffer, and the fluorescence image (black band) was then detected. The white band was derived from loading dye (bromophenol blue). To inhibit RNA degradation, 1 U of RNase OUT (O) was mixed into the samples but we checked RNase OUT did not affect the binding of RNA and VEGF165 by silver staining. Arrows indicate bands of RNA-protein complex.

To investigate the binding ability of VEGFA intronic G4_1 against VEGF165 protein *in vitro*, Electromobility shift binding assay (EMSA) was performed. The fluorescein-labeled VEGFA intronic G4_1 was used for binding analysis against VEGF165 protein. We examined VEGFA intronic G4_1 and its mutant mixed with a serial dilution of VEGF165 by non-denaturing-PAGE. As a result for fluorescence detection, we observed the band shift in the mixtures of VEGFA intronic G4_1 and 1000–2000 nM VEGF165 (Figure 1), indicating that VEGFA intronic G4_1 bound to VEGF165 protein whereas the band shifts were not observed at G to U mutant (VEGFA intronic G4_1 mutant). The band signals were weak but we observed it several times. For further analysis, we next performed the Surface Plasmon Resonance (SPR) analysis to determine the dissociation constant of G4-forming RNAs.

**Table 1:** Oligonucleotides used in this study.

Name	Sequence (5′–3′)	Length (mer)
VEGFA intronic G4_1	GGGGGCGGGAGCCAGAGACCAGUGGGCAGGG	31
VEGFA intronic G4_1 mutant	GUUUGCGGGAGCCAGAGACCAGUGGGCAGGG	31
VEGFA intronic G4_2	GGGGGCAGGGCGCAGGAGGGAGAGGGGG	28
VEGFA intronic G4_2 mutant	UUUUUCAGGGCGCAGGAGGGAGAUUUUU	28
VEGFA 5′ UTR G4	GGAGGAGGGGGAGGAGGA	18
VEGFA 5′ UTR G4 mutant	GGAUUAGUGUGAGGAGGA	18
PDGFA intronic G4_1	GGGGAAGGGGAGCUGGGGCGCAGCGGG	27
PDGFA intronic G4_2	GGGGUGCGGGGAGCGGGGAAGGG	23
PDGFB intronic G4_1	GGGCGCGGGGUUUGGGGUGGG	21
PDGFB intronic G4_2	GGGCACUCGGGUAGGGGGAGGACUAGGG	28
PDGFB intronic G4_3	GGGUUUGGUUGGGCACAGGGCACGGG	26

**Figure 2:**
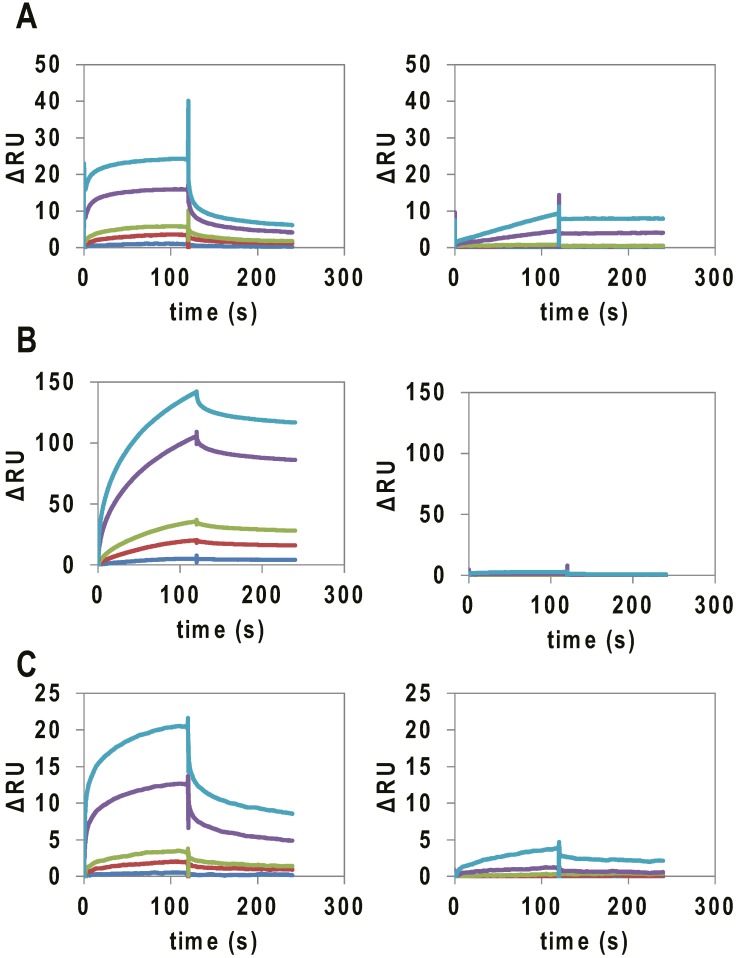
SPR analysis of the binding of G4-forming RNAs (concentration:10 nM (blue), 50 nM (red), 100 nM (green), 500 nM (purple), and 1000 nM (light blue)) to VEGF165. SPR signal of (**A**) VEGFA intronic G4_1 (left) and intronic G4_1 mutant (right); (**B**) VEGFA intronic G4_2 (Left) and VEGF intronic G4_2 mutant (Right); and (**C**) VEGFA 5′ UTR G4 (Left) and VEGF 5′ UTR G4 mutant (Right), to VEGF165 immobilized on a CM5 chip.

Binding assays of the RNAs to VEGF165 were performed by SPR. VEGF165 was immobilized on a CM5 chip via amine coupling, and 10–1000 nM of the RNAs were applied to the sensor chip. As controls, mutant and VEGFA intronic G4_1, G4_2 and VEGFA 5′ UTR G4, which were not expected to form G4 structures, were used (Table 1). In SPR analysis, a signal was observed for the binding of VEGFA intronic G4_1, G4_2 and VEGFA 5′ UTR G4 to immobilized VEGF165 (Figure 2), and the *K*_d_ values were calculated to be 141, 34, and 300 nM, respectively (Table 2). On the other hand, the mutants did not bind to VEGF165.

**Table 2:** Binding ability of G4-forming RNAs to VEGF165, PDGF-AA, and PDGF-BB.

Name	*K*_d_ (nM)		
	VEGF165	PDGF-AA	PDGF-BB
VEGFA intronic G4_1	140	20	40
VEGFA intronic G4_2	31	20	30
VEGFA 5′ UTR G4	300	60	110
PDGFA intronic G4_1	490	30	30
PDGFA intronic G4_2	200	30	30
PDGFB intronic G4_1	130	30	40
PDGFB intronic G4_2	150	20	30
PDGFB intronic G4_3	440	40	60

### 2.3. Evaluation of G4 RNA Structures by Circular Dichroism (CD) Spectroscopy

In the CD spectra measurements, the G4-forming RNAs had a negative peak at approximately 240 nm and a positive peak at approximately 260 nm, indicating that the RNAs adopt a parallel type G4 structure (Supplemental Figure S2). The mutant RNAs (mutant of VEGFA intronic G4_2 and VEGFA 5′ UTR G4), on the other hand, had neither the negative peak at approximately 240 nm, nor the positive peak at approximately 260 nm. The VEGFA intronic G4_1 seemed to have the 240 nm negative peak and 260 nm positive peak, this oligonucleotide formed unexpected parallel G4 structure that did not have the binding ability to VEGF165 protein. These results indicate that the G4-forming RNAs that are located within the 5′ UTR and the first intronic RNA region recognized VEGF165 with high affinity and that parallel type G4 structures are important for binding to VEGF165.

### 2.4. Binding Specificity of G4 RNAs Derived from Transcribed RNA to Heparin-Binding Proteins

We previously reported that parallel G4-forming DNAs that are located on *VEGFA*, *PDGFA*, and *RB1* promoters bound to the heparin-binding domain of VEGF165. The G4-forming RNAs derived from 5′ UTR and intronic RNA of *VEGFA* might form a parallel type G4 structure and bind to VEGF165. We expected that G4-forming RNAs might bind to other heparin-binding proteins. Therefore, binding analyses of the VEGFA G4-forming RNAs to PDGF-AA, PDGF-BB and Thrombin were performed by SPR. PDGF-AA, PDGF-BB and Thrombin were separately immobilized on a CM5 chip and the RNAs were applied. Signals indicating the binding of the G4-forming RNAs to PDGF-AA and PDGF-BB were observed, and the *K*_d_ values were calculated to be at the in the nanomolar level, indicating that the VEGFA G4-forming RNAs bind to PDGF-AA and PDGF-BB with an affinity similar to their binding affinity for VEGF165 (Table 2, Supplemental Figure S3). On the other hand, the binding signal to the Thrombin was not observed, indicating that the VEGFA G4-forming RNAs did not have the binding ability against Thrombin (Supplemental Figure S3).

### 2.5. Binding Abilities of G4 RNA from PDGFA and PDGFB pre-mRNA

The first introns of *PDGFA* and *PDGFB* also contain several potential G4-forming sequences. Of these sequences, two potential G4-forming RNAs located in the intronic RNA of *PDGFA* and three potential G4-forming RNAs within the intronic RNA of *PDGFB* were chemically synthesized in order to analyze their binding affinities against VEGF165, PDGF-AA, and PDGF-BB. SPR analysis indicated that these G4-forming RNAs bound to VEGF165, PDGF-AA, and PDGF-BB as well as the VEGF G4-forming RNAs (Table 2, Supplemental Figures S4, S5 and Table S1). The *PDGFA* and *PDGFB* RNAs also showed CD spectra typical of parallel type G4 structures (Supplemental Figure S6). These results suggest that parallel G4-forming RNAs might interact with the heparin-binding proteins with high ability.

Several functional G4-forming RNAs have been identified in telomeres as well as pre-mRNAs. In telomeres, non-coding RNA transcribed from subtelomeric promoter folds into a G4 structure to promote telomere heterochromatinization [20,21]. In pre-mRNA, G4-forming RNAs have been found in 5′ UTRs [22], IRESs [19], open reading frames [23], intronic RNA [24,25] and 3′ UTRs [26]. These G4 RNAs are involved in translation regulation and pre-mRNA processing, including splicing [24,25] and polyadenylation [26]. On the other hand, to the best of our knowledge, there has been no report on the biological function of G4-forming RNA on excised intronic RNA. In this study, we demonstrated that G4-forming RNAs that are located within intronic RNA regions could bind to VEGF165, PDGF-AA, and PDGF-BB with *K*_d_ of nanomolar range, suggesting that G4-forming excised intronic RNA can bind to the heparin-binding domains of the proteins. The heparin-binding domains have been identified in proteases, esterases, growth factors, chemokines, lipid-binding proteins, pathogen proteins, and adhesion proteins [27]. The function of these proteins is affected by heparin binding [28,29] suggesting that the G4-forming intronic RNA might be involved in controlling the function of heparin-binding proteins. Further studies such as cross-linking immunoprecipitation (CLIP) are needed to validate and investigate the actual interaction between G4-forming RNA and the protein in the cell.

The G4 RNAs identified here bound not only to VEGF165, but also PDGF-AA, and PDGF-BB. However, we have developed a sequence mutation method, which we termed as *in silico* maturation (ISM), to improve the function of aptamers based on genetic algorithms. Using ISM, we can improve the binding ability of G4-forming RNA by optimization of G-run and loop sequences because of the loop sequence in the G4 aptamer are involved in the binding to the target protein [30]. In addition, we succeeded to improve the binding ability, binding specificity, and inhibitory activity [31,32,33,34,35], therefore we believe that specificity of these G4-forming RNAs against VEGF165, PDGF-AA, and PDGF-BB would be improved using ISM.

## 3. Experimental Section

### 3.1. Materials

VEGF165, PDGF-AA, and PDGF-BB were purchased from R & D systems (Minneapolis, MN, USA). Human alpha-Thrombin was purchased from Haematologic Technologies Inc. (Essex, VT, USA). All RNAs were purchased from Greiner Japan (Tokyo, Japan) or Hokkaido System Science (Hokkaido, Japan).

### 3.2. Identification of Putative G4-Forming RNAs

Transcribed RNA sequences of *VEGFA*, *PDGFA*, and *PDGFB* were obtained from the UCSC genome browser [36] and putative G4-forming RNAs located on the transcribed RNAs were identified by the QGRS Mapper [18]. The synthesized RNAs were dissolved as 100 μM stock solutions in TE buffer (10 mM Tris-HCl, 1 mM EDTA, pH 8.0).

### 3.3. Electromobility Shift Assay (EMSA)

First, the chemically synthesized RNA oligonucleotides were labeled with fluorescein dye using the 5′ EndTag™ nucleic acid labeling system (Vector Laboratories, Burlingame, CA, USA). 0.8 nmol RNAs were used for fluorescein labeling and the labeling procedures were performed according to a prescribed protocol. After that, the ethanol precipitated RNAs were dissolved in 10 μL TE buffer.

Next EMSA was performed for evaluation of binding ability of RNAs. Fluorescein labeled RNAs were diluted into 2 μM in Tris-HCl potassium chloride buffer (50 mM Tris-HCl, with 100 mM KCl, pH 7.5) and folded at 65 °C for five min and then allowed to cool to room temperature for 30 min. Heat treated RNAs (f.c. 1 μM) and several concentrations of VEGF165 (f.c. 0.25, 0.5, 1, 2 μM) were mixed in Tris-HCl potassium chloride buffer. For inhibition of RNA degradation, 1 U of RNase OUT (Invitrogen, Carlsbad, CA, USA) was mixed into the samples then the mixtures were incubated at room temperature for 30 min. After incubation, ten microliters of the mixtures were electrophoresed on 15% polyacrylamide gel in TBE buffer, followed by fluorescein scanning the gel using Typhoon8600 (GE Healthcare, Little Chalfont, Buckinghamshire, UK).

### 3.4. Surface Plasmon Resonance (SPR) Assay

The binding affinities of the G4-forming RNAs for VEGF165, PDGF-AA, and PDGF-BB were analyzed at 25 °C on a Biacore T200 instrument (GE Healthcare). VEGF165 (in 10 mM acetate buffer, pH 6.0), PDGF-AA (in 10 mM HEPES, pH 7.0), and PDGF-BB (in 10 mM HEPES, pH 7.0) were immobilized on a CM5 sensor chip (GE Healthcare) by the amine coupling procedure, and the RNAs were injected over the surface. Prior to use, all RNAs were denatured in PBS buffer (137 mM NaCl, 2.7 mM KCl, 8 mM Na_2_HPO_4_, 2 mM KH_2_PO_4_, pH 7.4) at 65 °C for five min, and then allowed to cool to room temperature for 30 min. PBS served as both the running and aptamer dilution buffers. SPR signals were used for the construction of the dissociation constant (*K*_d_ value), and *K*_d_ was estimated by curve fitting using BIAevaluation software (GE Healthcare).

### 3.5. Circular Dichroism (CD) Spectroscopy

Circular dichroism (CD) spectra were recorded on a Jasco-820 spectropolarimeter (JASCO; Tokyo, Japan) using a quartz cell of 10 mm optical path length (Agilent Technologies, Santa Clara, CA, USA) and an instrument scanning speed of 500 nm/min. All RNA samples were diluted to 2 μM in PBS buffer. These RNA samples were then folded by heat treatment as described above. The CD spectra are representations of 10 averaged scans taken at 25 °C.

## 4. Conclusions

In this study, we identified new RNA oligonucleotides that formed G4 located within the 5′ UTR and first intron of *VEGFA* bind to VEGF165 with high affinity. Moreover, G4-forming RNAs located within *PDGFA* and *PDGFB* introns bound to PDGF-AA and PDGF-BB, respectively. Mutation analysis of G4-forming RNAs-VEGF165 interaction indicated that the G4 structure was important for binding to VEGF165. These results indicated that G4s in the pre-mRNA would be aptamers against the target protein that is encoded in the pre-mRNA. The potential G4-forming RNA sequences are located in RNA regions of many of the kinds of genes and the physiological function of the G4 RNAs has not been revealed. Taken together, our present research provides not only the methodology of G4-forming RNA aptamer identification, but also new insights on the physiological function of the G4-forming RNAs.

## Supplementary Materials

Supplementary materials can be accessed at: http://www.mdpi.com/1420-3049/20/11/19733/s1.
