# Supplementary material for: Identification of RNA Oligonucleotides Binding to Several Proteins from Potential G-Quadruplex Forming Regions in Transcribed Pre-mRNA
Source: Molecules. 2015 Nov 23;20(11):20832–40. doi: 10.3390/molecules201119733 (PMC6332122; doi:10.3390/molecules201119733)
Supplement: Supplementary file 1 [file molecules-20-19733-s001.pdf]

Supplemental Material: Identification of RNA Oligonucleotides Binding to Several Proteins from Potential G-Quadruplex Forming Regions in Transcribed Pre-mRNA

Taiki Saito, Wataru Yoshida, Tomomi Yokoyama, Koichi Abe and Kazunori Ikebukuro

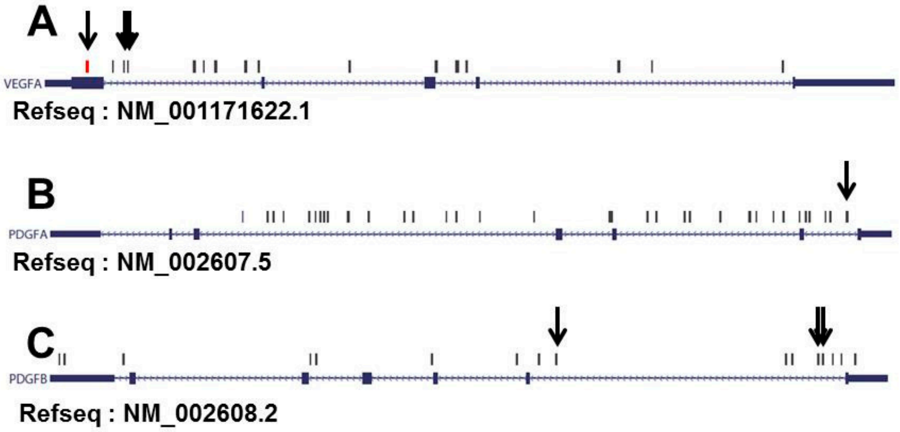

Figure S1. Map of the G4-forming RNAs in the (**A**) *VEGFA*; (**B**) *PDGFA*; and (**C**) *PDGFB* genes. The location of G4-forming sequences ($G_{\geq 3}N_{\geq 1}G_{\geq 3}N_{\geq 1}G_{\geq 3}N_{\geq 1}G_{\geq 3}N_{\geq 1}$; length ≤ 35-mer) are represented by black vertical bar. The G4-forming sequence in the 5′ UTR of *VEGFA* mRNA is represented by red vertical bar. The sequences used in this study are indicated by arrows.

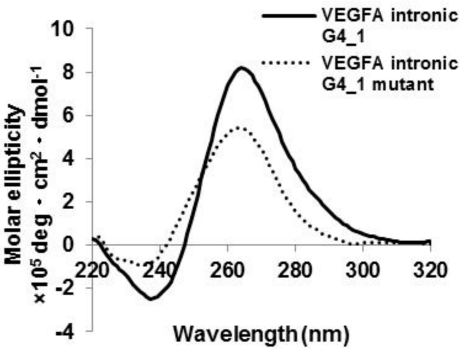

(**A**)

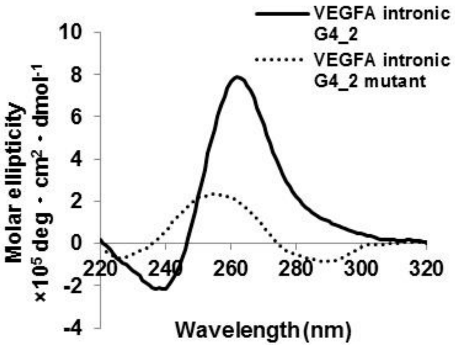

(**B**)

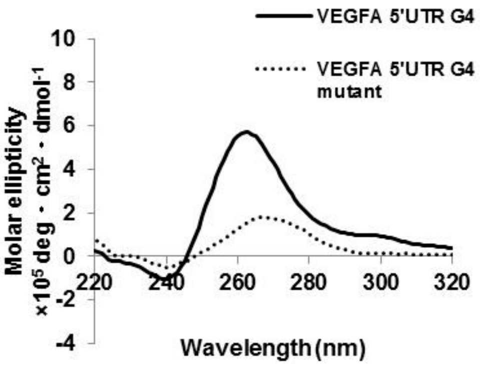

(**C**)

Figure S2. CD spectra of the VEGFA G4-froming RNAs. (**A**) VEGFA intronic G4_1 and VEGFA intronic G4_1 mutant; (**B**) VEGFA intronic G4_2 and VEGFA intronic G4_2 mutant; (**C**) VEGFA 5′ UTR G4 and VEGFA 5′ UTR G4 mutant, were analyzed.

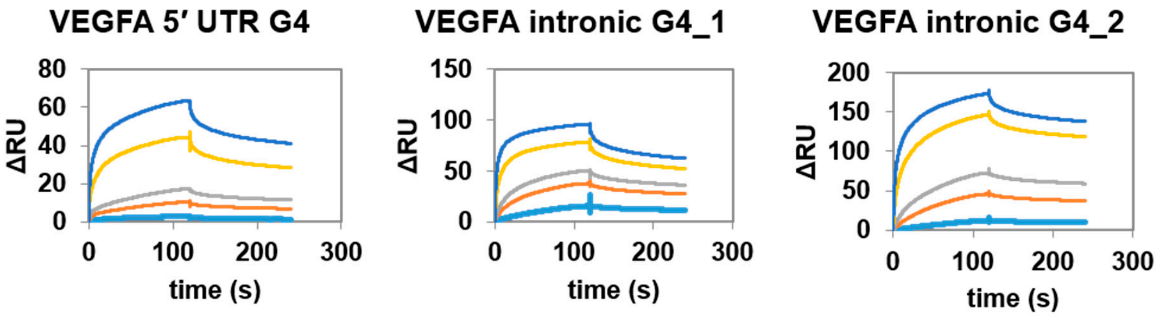

(**A**)

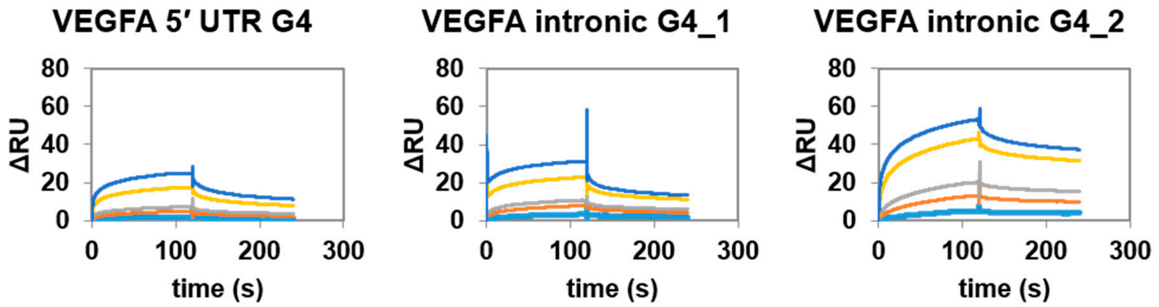

(**B**)

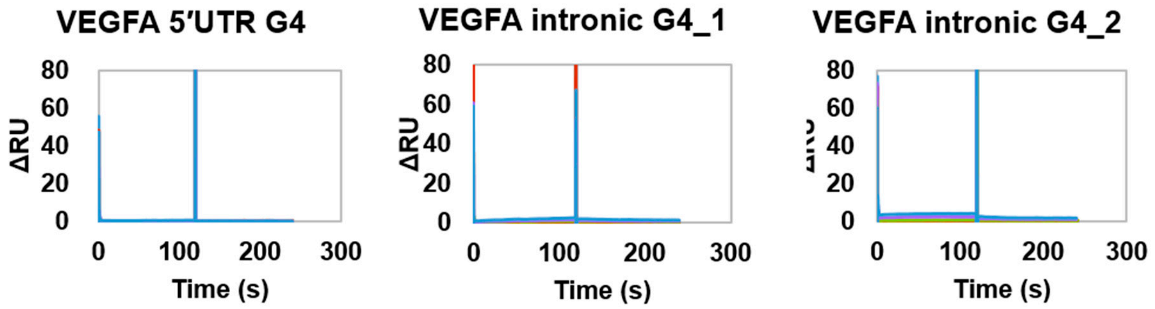

(**C**)

Figure S3. SPR analysis of VEGFA 5′ UTR G4, VEGFA intronic G4_1, and VEGFA intronic G4_2 to (**A**)PDGF-AA; (**B**)PDGF-BB and (**C**) Thrombin.

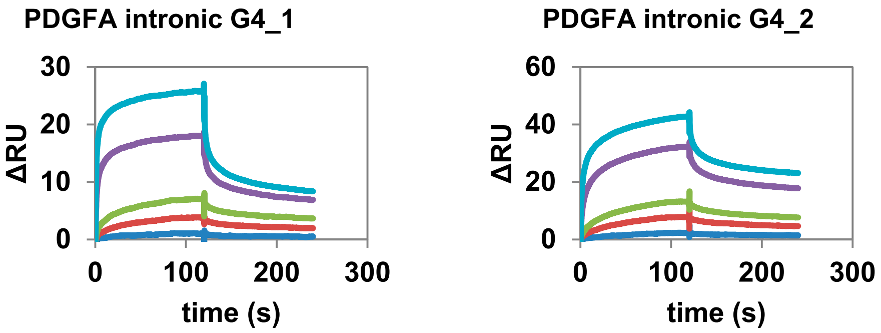

(**A**)

Figure S4. *Cont.*

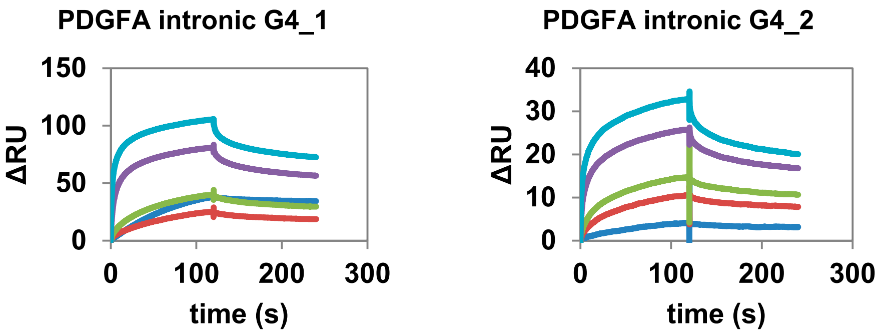

(**B**)

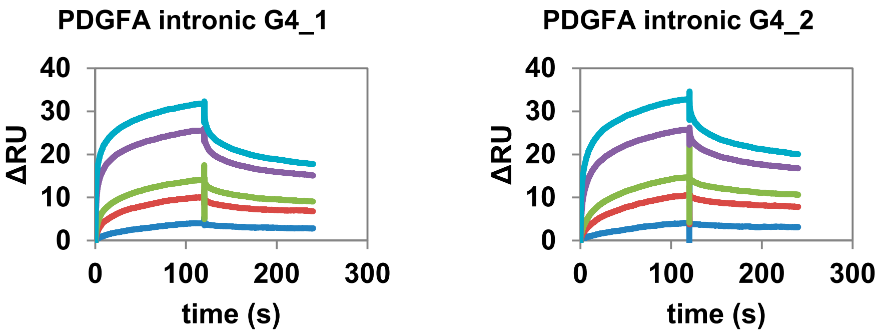

(**C**)

Figure S4. SPR analysis of PDGFA intronic G4_1 and G4_2 to (**A**) VEGF165, (**B**) PDGF-AA, and (**C**) PDGF-BB.

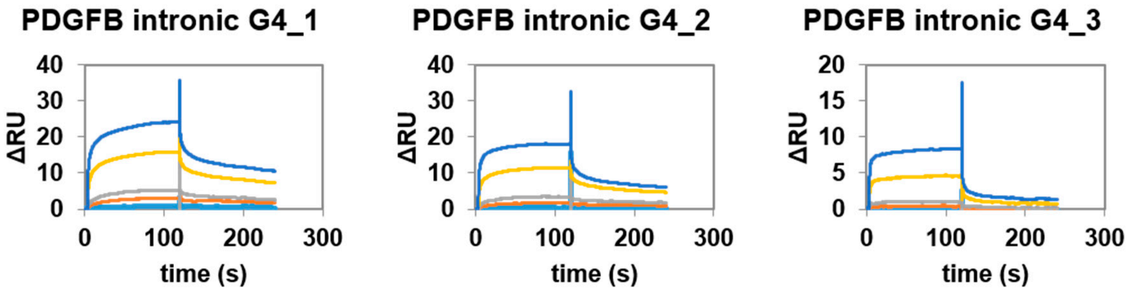

(**A**)

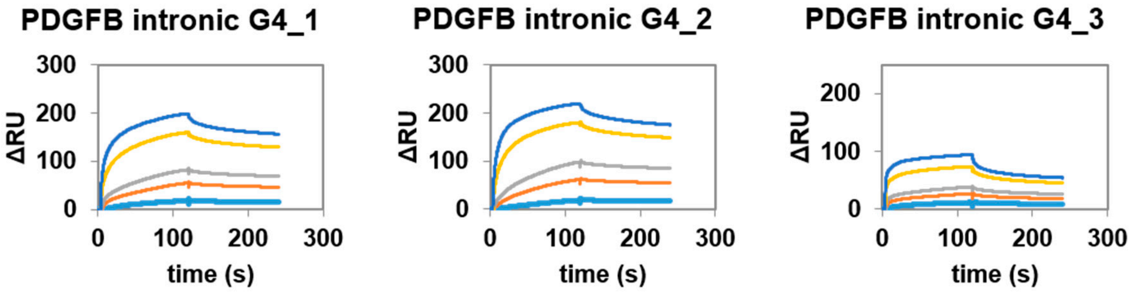

(**B**)

Figure S5. *Cont.*

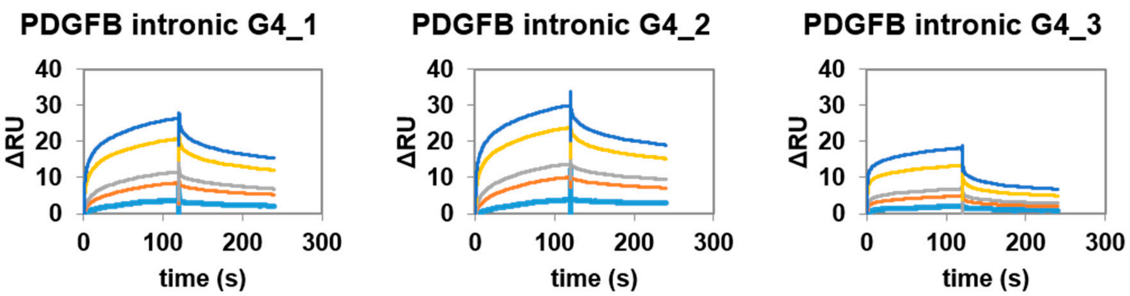

(**C**)

Figure S5. SPR analysis of PDGFB intronic G4_1, G4_2, and G4_3 to (**A**) VEGF165; (**B**) PDGF-AA, and (**C**) PDGF-BB.

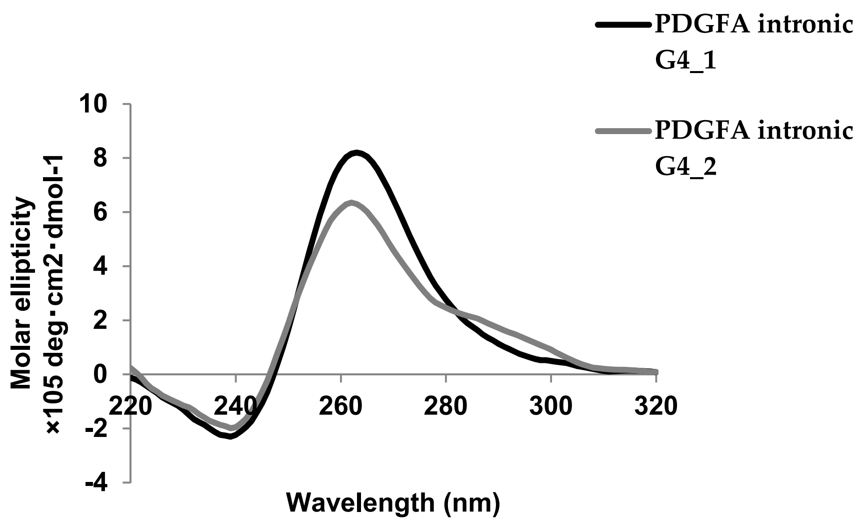

(**A**)

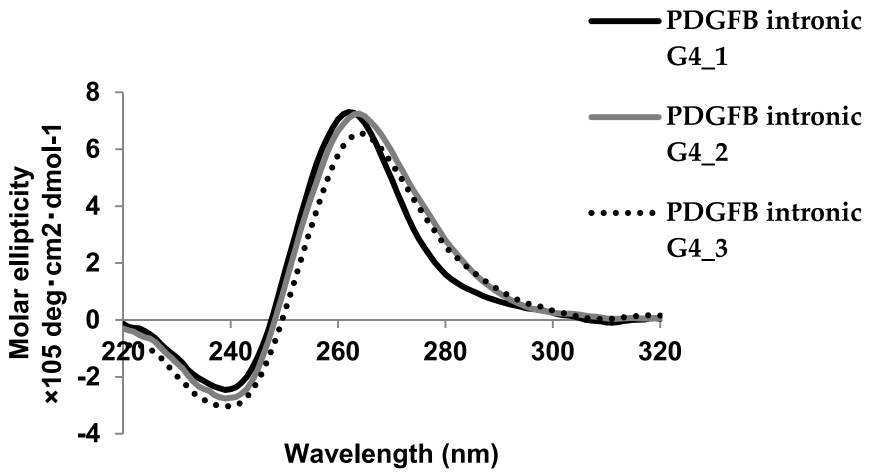

(**B**)

Figure S6. CD spectra of PDGFA intronic G4_1 and G4_2 (**A**) and PDGFB intronic G4_1, G4_2, and G4_3 (**B**).

Table S1. Kinetic value of G4-forming RNAs to VEGF165, PDGF-AA, and PDGF-BB.

**Protein**	**VEGF165**		
**Name**	k_{on} **(×10⁴ 1/Ms)**	k_{off} **(×10⁻³ 1/s)**	K_d **(nM)**
VEGFA intronic G4_1	4.6	6.6	140
VEGFA intronic G4_2	2.2	6.9	31
VEGFA 5′ UTR G4	1.2	3.7	300
PDGFA intronic G4_1	1.2	6.1	490
PDGFA intronic G4_2	3.4	6.8	200
PDGFB intronic G4_1	3.5	4.6	130
PDGFB intronic G4_2	4.5	6.5	150
PDGFB intronic G4_3	2.6	11	440
**Protein**	**PDGF-AA**		
**Name**	k_{on} **(×10⁴ 1/Ms)**	k_{off} **(×10⁻³ 1/s)**	K_d **(nM)**
VEGFA intronic G4_1	11	2.3	20
VEGFA intronic G4_2	5.3	1.0	20
VEGFA 5′ UTR G4	2.9	1.8	60
PDGFA intronic G4_1	6.4	1.8	30
PDGFA intronic G4_2	4.8	1.4	30
PDGFB intronic G4_1	5.7	1.5	30
PDGFB intronic G4_2	6.4	1.5	20
PDGFB intronic G4_3	7.9	3.3	40
**Protein**	**PDGF-BB**		
**Name**	k_{on} **(×10⁴ 1/Ms)**	k_{off} **(×10⁻³ 1/s)**	K_d **(nM)**
VEGFA intronic G4_1	6.3	2.5	40
VEGFA intronic G4_2	5.1	1.4	30
VEGFA 5′ UTR G4	3.8	4.3	110
PDGFA intronic G4_1	8.3	2.5	30
PDGFA intronic G4_2	8.5	2.3	30
PDGFB intronic G4_1	7.7	3.3	40
PDGFB intronic G4_2	8.4	2.5	30
PDGFB intronic G4_3	7.3	4.3	60
